# Photocatalytic degradation of organic pollutants using Trianthema Portulastrum leaf extract based CeO2 nanoparticles

**DOI:** 10.6026/97320630016765

**Published:** 2020-10-31

**Authors:** A Swetharanyam, R Kunjitham

**Affiliations:** 1 Research scholar, PG & Research department of chemistry, Department of Chemistry, Poompuhar College (Autonomous) Melaiyur - 609 107 (Affiliated to Bharathidasan University, Tiruchirappalli, Tamil Nadu- 620024), India; 2 PG & Research department of chemistry, Department of Chemistry, Poompuhar College (Autonomous) Melaiyur - 609 107 (Affiliated to Bharathidasan University, Tiruchirappalli, Tamil Nadu- 620024), India

**Keywords:** CeO2 nanoparticles, plant extract, dye degradation, antibacterial, antioxidant

## Abstract

Comparison of bio CeO2-Nps prepared using Trianthema Portulastrum leaf extract with chemical CeO2-Nps is of interest. The ultraviolet - visible, x-ray diffraction, HR - TEM, FT - IR, and photoluminescence studies were conducted with CeO2-Nps. UV-Maximum absorptionat 292 nm was completed using UV-visible spectrum. The HR–TEM images showed 38 nm bio CeO2-Nps with spherical morphology. This showed the polycrystalline character of CeO2-Nps similar to XRD data. The presence of metal oxide is confirmed by FT - IR analyses. The CeO2-Nps showed the potential photocatalytic activity for Acid black 1 color degradation after exposure to sunlight. Chem and bio CeO2-Nps have a degradation rate of 86.66 and 94.33%, respectively for acid black 1 dye. The synthesized CeO2-Nps are also evaluated for antibacterial and antioxidant activity. The bio CeO2-Nps has antibacterial activity for Pseudomonas aeruginosa (17 ± 0.56 mm) and Staphylococcus aureus (16 ± 0.24 mm) at low concentrations of 100 µl. The CeO2-Nps bio showed high inhibition of radical DPPH IC50 µg/ml, at 95.17 ± 21. Thus, we show that CeO2-Nps have environmentally friendly properties that are useful for dye degradation with antimicrobial and antioxidant activities.

## Background

The development of green chemistry to synthesize metal-based nanoparticles with extracts of different plants is gaining momentum in recent years [1]. Environmental impacts by bio nanoparticles are highly commended [2]. The plants have various types of phenolic and flavonoid compounds that help in nanoparticular formation [3,4]. It has been found that the extracts from various plants, such as Cataranthus roseus [6], Cocos nucifera [7], Beta vulgaris [8], Catunareg amspinosa [9] and Cyphomandra betacea were used to synthesize non - toxic nanomaterials [10]. The release of toxic substances affecting the environment by several industrial and research activities is evident [11]. Biosynthesized noble metal (Ag, Au... nanoparticles) are used for environmental friendly detoxification and elimination of harmful and deadly materials.[12,13]. The mechanism of •OH in biodegradation is known [14-16]. The formation π complexes as precursors of •OH adduct in hydroxylated by-products with γ-radiolysis is described [17-19]. The NPs play an important role in the removal of organic and inorganic contaminants [20-22]. The bio CeO2-NPs synthesis is environmentally friendly and non-toxic [23- 24]. CeO2-NPs are effective alternatives to degrade dyes and other pollutants [25-26]. Therefore, it is of interest to document the photocatalytic degradation of organic pollutants using bio (Trianthema Portulastrum leaf extract) CeO2 nanoparticles in comparison with the chemical CeO2 naoparticles.

## Methodology

Fresh Trianthema portulastrum leaves were collected from chidambaram rural areas, Tamil Nadu, India. Cerium chloride (CeCl3) (99.9%) was obtained and used as received by Sigma - Aldrich, Bangalore, India. Staphylococcus aureus (ATCC 6538) and Pseudomonas aeruginosa (ATCC 9022) were obtained from the microbial culture collection, the Institute of Microbial Technology in Chandigarh, India,. Petri plates were selected with a diameter of about 32 cm and a thickness of 2 cm. All other used reagents are of analytical quality. 

## Preparation of CeO2 Nps using chemical method:

Cerium chloride (CeCl3) was used without further purification as they were received. CeO2-Nps were developed using sole-gel processes [27]. 3.72 g ceriam chloride salt taken in 10 ml of deionized water, and ammonia was added drop-by-side until its pH attained 10. The continious stirring for another two hours until all the precipitation was over. Filters wash and dry the precipitates overnight. The powder was then calcinated at a temperature of 400°C for two hours in an oven.

## Preparation of CeO2 Nps using plant extract method:

10g Trianthema Portulastrum leaf was powdered and mixed with 100 mL of water at 80°C. The extract of the leaf was filtered with Whatman No. 1. In 100-ml Erlenmeyer, it was preserved for further use at room temperature. 1:2 v/v CeO2 were prepared using 10 ml CeCl3 (contains 3.72g) and a 5 ml leaf extract. At a temperature of 85°C, the mixture was agitated for 4 hours. The yielding of CeO2-Nps observed yellowish-brown color. Also, the precipitate was dried for 4 hours at 400°C.

## Characterization of CeO2 Nps

TEM images of metal oxide nanoparticles were obtained using a transmission electron microscope (PHILIPS CM200 model) at an operating voltage of 20-200kv with resolution: 2.4 Ao. XRD spectra were recorded on the X'PERT PRO model X-ray diffracto-meter from Pan Analytical instruments operated at a voltage of 40 kV and a current of 30 mA with Cu Ka radiation. The FT-IR spectra of powdered CeO2 were mixed with KBr pellets and are recorded in the 4,000 - 400 cm-1 range on a Shimadzu FTIR-8400s. To investigate optical responses and compute the bandgap, the synthesized CeO2-NP samples have been subject to UV-vision spectroscopy (Shimadzu UV 1650). The energy for the nanoparticles optical band gap is calculated using the Tauc relation based on the absorption spectrum of the nanoparticles:

αhv = A (hv-Eg)1/2

Where α is a coefficient of optical absorption, the photon energy is hv, eg is a bandgap direct, and A is a constant that is energy-dependent.

Size of the synthesized CeO2 Nps can be calculated by applying the following equation [28]:

D = 0.9λ/βcosθ (Scherrer equation)|

If D is of crystalline size, k is of a shape factor (K= 0.9 in this work), θ is of Bragg angle, β is of full width at half-maximum and λ is of wavelength of X - ray incident. Photoluminescence ( PL) behavior was found at room temperature by FLUOROLOG-3.

## Photocatalytic activity:

The photocatalytic activities of Chem CeO2-Nps and plant mediated CeO2-Nps were analyzed using the reactions of acid black 1 dye under-stimulated sunlight irradiation. In that experiment, 100 ml of 0.2 g of fine powder catalyst (Chem CeO2-Nps and bio CeO2-Nps) and 3 x 10-4 M aqueous acid black 1 dye were taken. Photocatalytic measurement time ranged between 0 and 80 minutes. The suspension allowed the adsorption to stir in the dark for 10 min to achieve the adsorption-desorption balance between the dye and nanoparticles. Subsequently. The suspension was placed under sunlight and read every ten minutes up to 80 minutes.

On the catalyst surface, the proportion of acid black 1 was estimated following the following ratio [29]:

Degradation (%) = C0-Ct/C0 x 100

where C0 is the initial absorption and Ct is the absorption after different intervals of time.

## Antioxidant studies using DPPH method:

1'1-diphenyl-2-picryl hydroxyl radical methods, as reported on Das et al. [30], have been tested in Trianthema Portulastrum leaf extract, Chem CeO2-NP's, and Bio CeO2-Nps. Added to 0.1 mM methanol DPPH radical solution in equal volume, the different concentrations of (25/50/100/125/250/500 μg/ml) sample solution were provided. The reaction mixture was incubated for 60 minutes at room temperature. The mixture has been measured for the optical intensity of 517 nm, which provides antioxidant activity. Ascorbic acid was used for the calibration of the resulting activity as standard. The radical scavenging activity (RSA) percentage of the sample was calculated using the following equation:

% DPPH radical scavenging = (Absorbance of control - absorbance of test sample)/(Absorbance of control) x 100

## Antibacterial activity:

Antibacterial properties of fresh leaf extract and prepared nanoparticles biological and chemical method has explored by using disc diffusion technic [31]. It has been studied using the clinical isolation of bacterial cultures Gram-positive bacteria and Gram-negative bacteria Staphylococcus aureus and Pseudomonas aeruginosa as well. Dissolved nutrient agar was swept into the bacterial suspension, poured through sterile swabs of cotton, and produced with the help of an adjustable cork borer made from stainless steel. At 35°C for 48 hours, the plates were incubated. Ciprofloxacin is used as a positive control and the 50 µl and 100 µl Trianthema Portulastrum leaf extracts, the Chem CeO2-Nps and bioCeO2-Nps were added. Table 3(see PDF) shows the inhibition zone in diameter (mm).

## Statistical analysis:

The results were evaluated statistically by sigma plot 12.5; an average value for three different replications and a standard error (SE) was determined.

## Results and Discussion:

**Figure 1:**
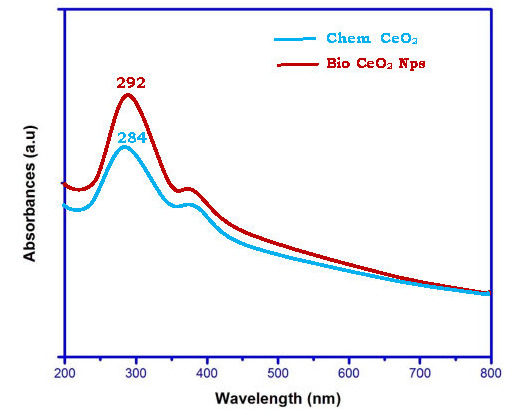
UV–visible spectra of Chem CeO2-NPs and biosynthesized CeO2-NPs

Chemical CeO2-Nps and bio CeO2-Nps are measured using the optical absorption (Figure 1). Chem CeO2-Nps and biosynthetic CeO2-Nps at 284 and 292 nm absorption peaks are observed and all these values are red shifts relative to the absorption maximum (284 nm) of the Chem CeO2. Qaisar et al have shown similar absorption peaks (at 315 nm) for bio CeO2-Nps [32]. The trianthema Portulastrum extract is comprised of phytochemicals that serve as a cap and reduction agent and also cause the UV absorption point to shift. The absorption position was suggested to depend on the size and shape of the particle in CeO2-NP. The UV - visible absorption potential of the CeO2-Nps is correlated with the bandgap energy, differentiating between CeO2-Nps in different forms. Tauc's equation is used to compute the gap of synthesized CeO2-Nps [33].

**Figure 2:**
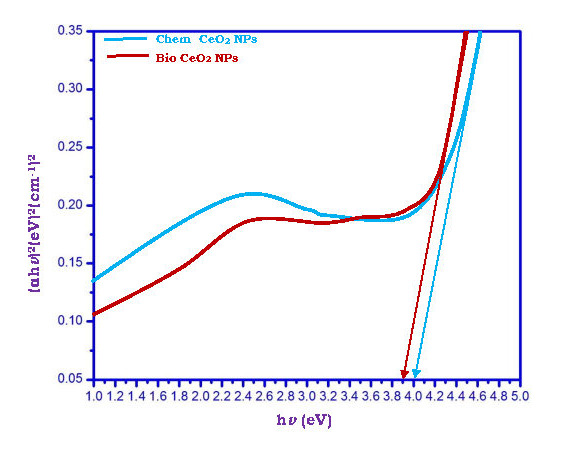
Band gap energy Chem CeO2-NPs and biosynthesized CeO2-NP

Chem CeO2-NP and bio CeO2-NP band gap energy values have been identified 4.00 and 3.90 eV respectively (Figure 2). The band gap of the biologically synthesized CeO2-Nps can be seen to be smaller than the Chem CeO2-Nps. The powerful interaction between CeO2 and Trianthema portulastrum leaf extract phytochemicals (flavonoids and proteins) allows for a faster process for recombining electrons and has resulted in a reduction in band gap for bio CeO2 Nps. For CeO2-NP-biosynthesised, the observed band gap value 3.90 eV is appropriate for photocatalytic and antibacterial activities, which involve electron-exciting formation.

**Figure 3:**
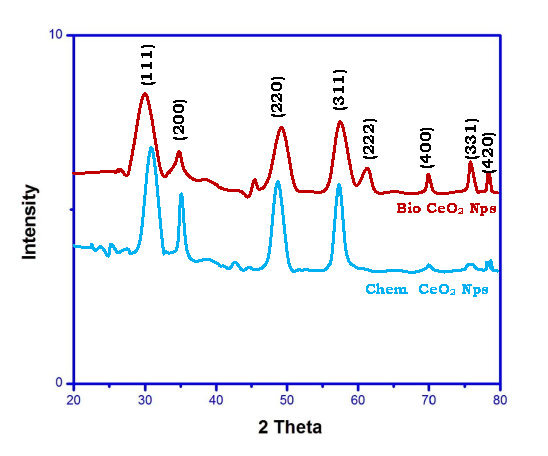
X-ray diffraction pattern of Chem CeO2-NPs and biosynthesize CeO2-NPs

Figure 3 shows the patterns of X-ray diffraction of chemicals CeO2-Nps and bio CeO2-Nps with various concentration of Trianthema portulastrum leaf extract. The sharp, intense diffraction peaks show Crystal structure and purity. The cubic structure of the CeO2-Nps (Jcpds no: 043-2002) is the most responsive Brags Peaks that can be reported with the Miller Index (111), (200), [220], (400), (331) and [422] [34-35]. In determining the average crystallite sample size, the Scherrer formula has been used. Figure 3 shows a crystal size of 78 nm for Chem CeO2-Nps.With an increasing percentage of Trianthema portulastrum leaf extract; the crystal size decreases for bio CeO2-Nps and is found to be 34 nm. Bio CeO2-Nps are observed to have a minimum crystallite size due to their quantum confinement effect.

**Figure 4:**
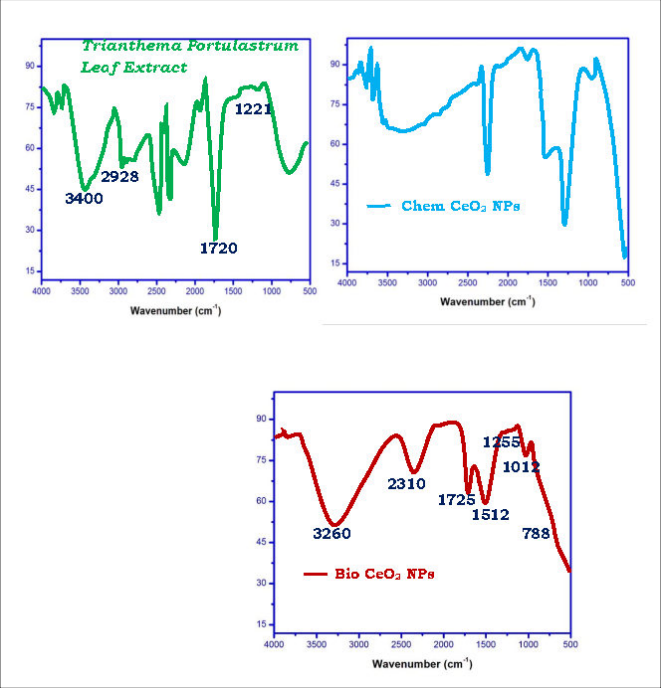
FT-IR spectrum of Trianthema portulastrum leaf extract, Chem CeO2-NPs and biosynthesized CeO2-NPs

The FT - IR spectroscopy helps to detect leaf extract bio-molecules attached to the CeO2 surface. FT - IR spectra for Trianthema Portulastrum dried leaf extract, Chem CeO2-Nps and CeO2-Nps bio are displayed in Figure 4a-c. Figure 4a shows the peaks and their assignments. FTIR spectroscopy illustrated absorption peaks at 3400, 2928, 1720, 1221 cm-1 were reproduced in the extract of Trianthema portulastrum leaf. The absorption band of O-H stretching vibration appears at 3400 cm-1. The absorption bands at 2928 and 1720 cm-1 is due to aldehydic C-H stretching and C=O vibration, respectively. 1231 cm-1 is due to C-N stretching vibration. Bio CeO2-Nps shows FT - IR peaks at 3260, 2310, 1725, 1512, 1255, 1012 and 788 is due to presence of free O - H attachment [36- 37], CH vibration, NH primary amines, CH2 bond, CH3 is due group, vinyl group and C - O stretching mode vibration [38]. The leaf extracts contain flavonoids that are potent reducing agents that can reduce cerium chloride heptahydrate salt. These flavonoids act as surfactants and are fixed to the CeO2-NP surface, and by electrostatic stabilization, they stabilize CeO2-NP's. As a result, Trianthema portulastrum leaf extract has a dual function to reduce and stabilize CeO2-Nps.

**Figure 5:**
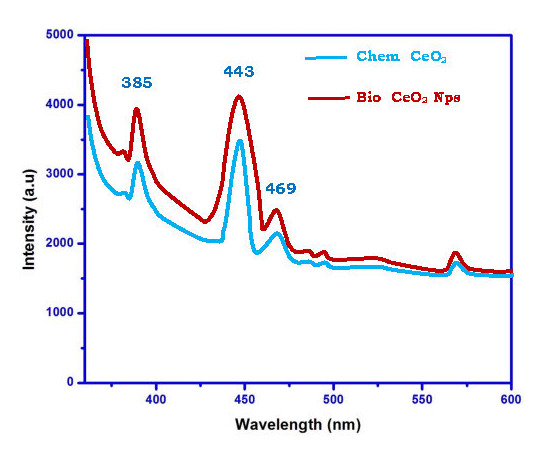
Photoluminescence spectra of Chem CeO2-NPs and biosynthesized CeO2-NPs

Photoluminescence spectroscopy (PL) usually explores the efficiency of the migration and transmission of charging carriers and also the chance of electron-hole pairs in metal oxide [39]. In this research, Photoluminescence spectrum is used to collect significant evidence about surface defects, oxygen vacancies and surface conditions which may sulphurise the impact of the photocatalytic response. The Chem CeO2-Nps and bio CeO2-Nps show room temperature PL spectrum in Figure 5. The two samples show similar peak positions but vary in intensity. With increasing leaf extract Trianthema portulastrum percentage the PL intensity increases. The synthesized CeO2-NP emission spectrum includes three peaks of 385,443 and 469 nm, which reflecting the near-band emissions one violet and two blue emissions. Excitonic recombination is the result of the Chem CeO2-Nps PL emittance peak at 389 nm. It is due to the transitions of 5d–4f of Ce3+ from ground state 2D(5d1) to state 2F5/2 (4f1) [40]. At 443 and 469 nm, the emission peak is related to oxygen vacancies [41-42]. The bio CeO2-Nps has a blue - shift at 443 nm and 469 nm compared with the chem CeO2-Nps.The blue emission peak lies at 443 nm due to the transition from the oxygen vacancy. The oxygen defects in bio CeO2-Nps thus support to connect the photo - induced electron easily in excitons. This shows that the intensity of PL has increased. The enhanced PL shows the intensity of bio CeO2-Nps' good crystalline nature and shows desirable catalytic properties.

**Figure 6:**
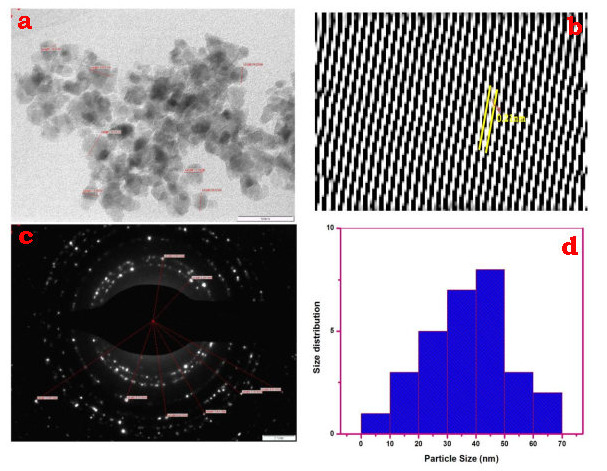
(a) HR-TEM image; (b) lattice fringe; (c) SAED pattern; (d) particle size of biosynthesized CeO2-NPs

**Figure 7:**
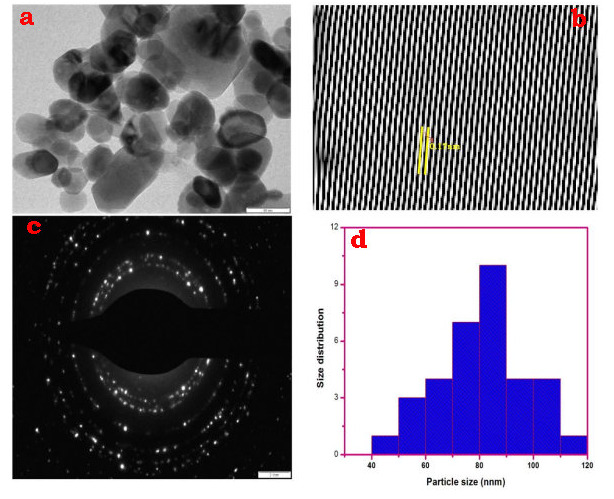
(a) HR-TEM image; (b) lattice fringe; (c) SAED pattern; (d) particle size of Chem CeO2-NPs

The morphological and particulate sizes of the synthesized CeO2-Nps are demonstrated by high - resolution transmission electron microscopy (HR-TEM). The Figure 6a&Figure 7a show typical TEMs obtained with CeO2-Nps prepared using trianthema Portulastrum extract and Chem CeO2-Nps.Synthesized CeO2-Nps have a morphology of almost cubic nanocrystals. In Figure 6-Figure 7 the histogram showing the distribution of particle size. The histogram in the bioCeO2-Nps and chemicals CeO2-Nps is narrower in width and the mean particle size is 38 and 82 nm.The particle size seen in HR-TEM is less than the dynamic light scattering value. The electron (SAED) pattern selected for the area is confirmed with the crystal plane nature of a bio CeO2-Nps, with the bright-circulated spots that correspond to the following (1 1 1), (2 2 0) (2 2 1), (2 2 2 2), (4 0 0), (3 3 1) and (4 2 0). The SAED pattern of crystalline impurities shows no other rings [43-44].

## Photocatalytic activity:

CeO2-Nps are environmentally friendly among many rare earth elements, due to their ecologically based photocatalytic application. Industrial waste contains various types of toxic and organic dyes released into water bodies. It has a major environmental impact. All dying agents are organically stable. The colors of acid black 1 dye in both oxidized and reduced shapes are different, so it is picked for the study.

**Figure 8:**
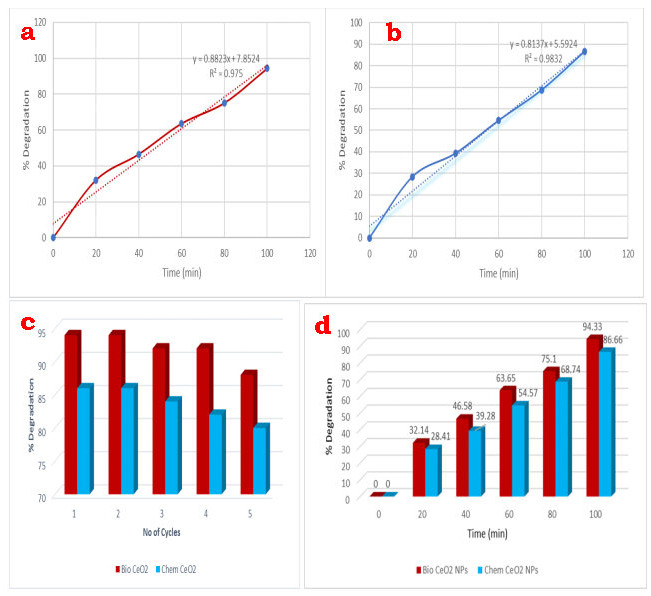
(a & b) Rate constant (K) and regression (R2); (c) Reusability of biosynthesized CeO2-NPs and Chem CeO2-NPs; (d) % degradation of acid black 1 dye compared to the biosynthesized CeO2-NPs and Chem CeO2-NPs

For chemical CeO2-Nps and bio CeO2-NP, photocatalytic activity is conducted to investigate the degradation of an aqueous acid black 1 dye solution by open-air sunlight. In Figure 8a-d you can see the catalytic degradation of the dye. The spectrum UV - Vis is recorded at different intervals 0, 20, 40, 60 and 80 min, between 200 and 800 nm. If it is acid black 1 dye, the peak UV absorption at 345 and 615 nm indicates that the dark blue of the dye becomes a colorless due to electron transition. The bands of 615 nm show that, owing to the catalytic effectiveness Chem CeO2-NP and the bio CeO2-N Ps, 86.66 and 94.33% of dye are exactamente 80 minutes degraded (Table 1 - see PDF). When the catalyst is added, the increased reduction rate is observed. This refers to the potential redox enhancement of the electron movement process between beneficiary and recipient. Bio CeO2-Nps act as an effective redox catalyst with an electron relay effect. The size of metal nanoparticles plays a major role in catalytic reductions, while the size of bio CeO2-Nps has decreased that promotes reactant adsorption on the catalyst surface and simplifies degradation. This will greatly improve the efficiency of the catalyst by increasing the particle surface area. Table 2(see PDF) shows reusability efficiency of bio and chem CeO2, upto two cycles there is no decrease in percentage degradation of acid black 1 dye.

## Mechanistic pathway of dye degradation:

The various quantities of oxygen vacancies show that photocatalytic results are different. It further suggests that significantly more oxygen vacancies will require quick recombination of electron holes and thus decrease photocatalysis for Chemical CO2-Nps [45]. The difference in photocatalytic activity has highly been linked in accordance with concentration errors on the nanoparticles surface [45]. They also showed that surface defects have been increased as the particle sizes decreased and photocatalytic activity increased. The present study shows high photocatalytic activity in bio-synthesized CeO2-Nps with the smallest particle size attributable to the high separation capacity of the photo generating chargers, large specific areas, and increased absorption of light. Based on these, the possible photo-degradation of the Acid black 1 dye over the UV-radiated CeO2-Nps is shown in Figure 8.

The above reaction stages allow electrons (e-) to be excited into the conductivity band (CB) by sunlight when the bi-synthesized CeO2-NP is radiated by the same number of holes (h+) in the VB. Photo-initiated holes react reasonably with Acid black 1 or attach to the surface H2O or OH - bound to provide a solid oxidant OH - radical species. It is suggested that the produced electron binds to O2 adsorbed to produce O2.- .This means that H+ produces HO2., which leads to radical OH• from the trapped electron. Therefore, the Acid black 1 dye could be degraded by produced reactive species such as OH⋅, HO2⋅, and O2.-

## Kinetic studies:

The kinetics of photocatalyst organic degradation in pseudo-first order is described elsewhere [46].

In (C0∕Ct) = -kt 

Where k is the apparent reaction rate constant, C0 is an initial concentration of aqueous Trypan blue, t is a time of reaction and C is an aqueous Acid black 1 color at a time of reaction t. Bio-synthesized CeO2-Nps and Chem CeO2-Nps are investigated and the kinetics of photodegradation of Acid black 1 is presented in Figure 8ab. A pseudo-first-order rate equation determines the rate constant (K) for Acid black 1 dye degradation using synthesized CeO2-Nps.The graph In (C0 / Ct) is a rate constant of bio and chemically synthesized CeO2 Nps 7.8524 and 5.5924 min-1 based on the irradiation duration. Also, 0.9832 and 0.9750 for Chem CeO2-Nps and CeO2-Nps bio are also determined for the fitting correlation coefficient (R2). Finally, C0/Ct decreased with time increasing and vice versa. With the increase in time, the percentage of degradation is increased (Figure 8d). As a result, BioCeO2-Nps demonstrated an improved photocatalytic effeciancy in Acid black 1 dye than Chem CeO2-Nps and other literature values.

## Antioxidant activity of Synthesized nanoparticles by using DPPH method:

**Figure 9:**
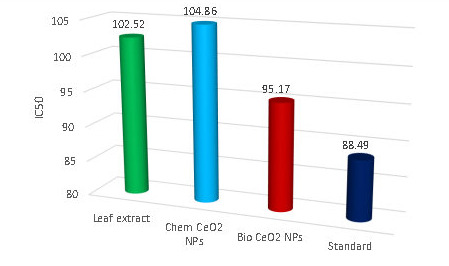
DPPH free radical assay of Trianthema portulastrum leaf extract, ChemCeO2-NPs and biosynthesized CeO2-NPs

DPPH Radical Trianthema portulastrum leaf extract scavenging activity Chem CeO2-Nps and CeO2-Nps are measured at various concentrations of (25/50/100/125/250/500 µg /ml) for standard ascorbic acid. By changing DPPH color, from the initial blue/purple solution to a yellow the reduced activity of Trianthema portulastrum leaf extract, bioCeO2-NP, and chemCeO2-Nps is determined. The percentage of DPPH inhibition is shown in Figure 9 & Table 3 (see PDF). For Trianthema portulastrum leaf extract, chem CeO2-Nps bio CeO 2-Nps, and the standard, the calculated halve maximum inhibitory concentration (IC50 µg / ml) values shall be 102.52, 104.86, 95.17 and 88.49. When IC50 µg / ml values are lower, the potential for extract antioxidant activity is higher. In comparison to Trianthema portulastrum leaf and chemical CeO2-NP, the study of DPPH scavenging activity has seen the greatest inhibition in bio CeO2-Nps.This result is following Fatemeh et al. studies, which have demonstrated the antioxidant activity of Ceratonia siliqua extract plants using bio CO2-Nps [47]. Moreover, the results of Krishanaveni et al. were comparable by the use of Clitoria ternatea bio CeO2-Nps. Antioxidant activities might be related by the presence of flavorous, alkaloides in the extract of Trianthema portulastrum leaf. This means a reduction in antioxidant activity may result in a reduction in the metabolite concentration of plants during nanoparticular formation. The surface area of cerium oxide is large, which means more plant chemical substances are added to the active surface. As a result, the shell response phenomenon in the extract of Trianthema Portulastrum leaves is elevated by bio CeO2-Nps (due to an adsorbed antioxidant moiety on the surface).

## Antibacterial activity by using disc diffusion method:

**Figure 10:**
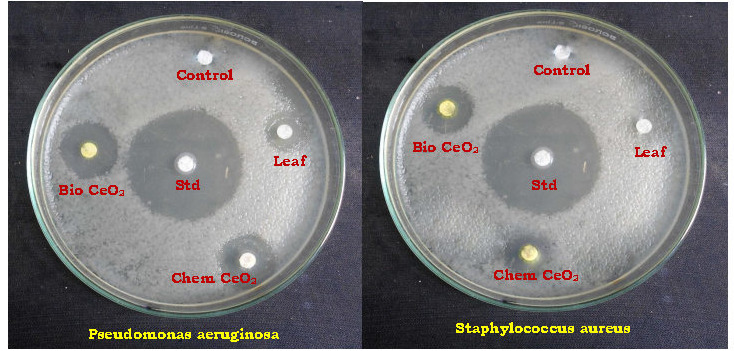
Antibacterial activity of Trianthema portulastrum leaf extract, Chem CeO2-NPs and biosynthesized CeO2-NPs against Pseudomonas aeruginosa and Staphylococcus aureus

Bacterial inhibition of Trianthema portulastrum extract, ChemceO2-NP and bio CeO2-Nps are analyzed and the area of inhibition is measured for Gram-positive Bacteria (Staphylooccus aureus) and Grass negative Bacteria (Pseudomonas aeruginosa) at 100 µl (Figure 10). Table 4(see PDF) shows the diameter of the inhibition zone (mm). The bio CeO2-Nps (17±0.56 & 16±0.24) exhibit improved bacteriocidal effeciancy than Chem CeO2-Nps (14±0.23 & 11±0.57) and Trianthema portulastrum leaf extracts (10±0.08 & 06±0.41) against Pseudomonas aeruginosa and Staphylooccus aureus. Particle size and surface area are known to play a key role in their connection with biological cells or to produce secondary damaging products. Due to their size and wide surface area, CeO2-Nps produce electronic effects. These electronic effects improve nanoparticles' coupling quality with the microbes CeO2-Nps can therefore easily be attached and inserted into the bacteria in the cell membrane [48]. The above mechanisms show that bio CeO 2-Nps have higher antibacterial activity in comparison with the leaf extract of Trianthema portulastrum and Chem CeO2-Nps. The increased inhibitory activity of bio ceO2-Nps depends not only on the size of nanoparticles and their surface but also on the capping agents (proteins).

## Conclusion:

The bio and chemical CeO2-Nps were synthesized, evaluated, characterized and compared for the photocatalytic degradation of organic pollutants. We show that CeO2-Nps degrades acid black 1 coloring under sunlight in a photocatal syst system. Photocatalyst bio CeO2-Nps exhibited excellent photocatalytic degradation under visible light irradiation of 94.33%. We also show that the bio CeO 2-Nps is have antibacterial activity. Data show that bio CeO2-Nps is associated with various biological and medical applications.
